# The Neuroanatomical Correlates of Dyspnea: An Activation Likelihood Estimation Meta-Analysis

**DOI:** 10.3390/neurosci6020036

**Published:** 2025-04-17

**Authors:** Christoph Müller, Jens Kerl, Dominic Dellweg

**Affiliations:** 1 Department of Internal Medicine, Lahn-Dill-Kliniken, 35578 Wetzlar, Germany; 2 Department of Internal Medicine, University of Marburg, 35037 Marburg, Germany; 3 Sleep Laboratory, Fachkrankenhaus Kloster Grafschaft, 57392 Schmallenberg, Germany; 4 Department of Pulmonology, Pius-Hospital Carl von Ossietzky University of Oldenburg, 26121 Oldenburg, Germany

**Keywords:** dyspnea, functional neuroimaging, activation likelihood estimation meta-analysis, interoception

## Abstract

The sensation of dyspnea is related to various cardiopulmonary and neuromuscular diseases and is characterized by its sensory and affective qualities. Although there is a vast number of studies investigating its pathophysiology, less is known about the neuroanatomy of dyspnea perception. An activation likelihood estimation (ALE) meta-analysis of 13 studies investigating different breathing challenges using either PET or fMRI was performed to demonstrate the neuroanatomical correlates of dyspnea perception. The ALE meta-analysis was performed using the GingerAle software 3.0.2 and was displayed with the Mango software 4.1. Synthesizing the results of all included studies, clusters involving the insula and cingulated cortex in both hemispheres were observed. Subgroup analysis for the restrained breathing condition revealed activation involving the right and left cingulate cortex and left anterior cingulate cortex. For the loaded breathing condition, statistically significant activation was found for the postcentral gyrus, the superior temporal gyrus, and the right thalamus. The combined ALE map for both conditions showed activity patterns in the right cingulate cortex, the right insula, and the right thalamus. This ALE meta-analysis demonstrates that two separate neuronal pathways related to either the affective or intensity domain are involved in the central processing of dyspnea perception.

## 1. Introduction

Dyspnea is a multi-dimensional sensation which occurs as the result of an imbalance between the demand and the ability to breath [1]. It is a common symptom affecting about 50% of patients in the acute hospital setting and can be due to cardiac, pulmonary, neurological, and muscular disease [2]. The terminology of dyspnea meaning disordered (gr. dys) breathing (gr. pnea) does not account for its sensory, cognitive, and affective dimensions and neglects the social consequences for patients during everyday life. A more comprehensive definition is provided by the American Thoracic Society which describes dyspnea as a “subjective experience of breathing discomfort that consists of qualitatively distinct sensations that vary in intensity (…) and derives from interactions among multiple physiological, psychological, social, and environmental factors, and may induce secondary physiological and behavioral responses” [3]. While the sensory domain of dyspnea is related to the intensity of the perception, the affective dimension is associated with the feeling of unpleasantness and fear. Different challenges can induce dyspnea including breathing against a resistive airway, chest wall constriction, or breathing of hypercapnic gas mixtures [4]. These conditions may result in specific sensory qualities including chest tightness (constriction of airways), effort (afferents from respiratory muscles), or air hunger (not getting enough air). While conditions with resistive loads may cause a sensation of increased effort (work of breathing) by stimulating respiratory motor afferents, restricted breathing may rather lead to the sensation of air hunger sensed by chemoreceptive or pulmonary afferents [5]. Both the sensory and affective dimension of dyspnea are related to distinct neuroanatomical pathways. The sensory or intensity domain of dyspnea corresponds to the somatosensory information which is sent to the brainstem and is then relayed from the ventroposterior thalamus to the primary and secondary somatosensory cortex. The affective processing of dyspnea begins with afferents of airway and lung receptors sent to the brainstem via the vagal nerve from where information is sent to the amygdala and dorsal areas of the thalamus. These send projections to the insula and cingulate cortex where an awareness of the affective domain of dyspnea emerges [6]. While breathing is usually unconscious, its aware perception is considered to be a warning signal which prompts the individual to adapt its behavioral response. The awareness of inner states can, therefore, be considered as a basic form of consciousness which enables the organism to correct for somatovisceral imbalances and to regain homeostasis [7].

The neuroanatomical correlates of inner perceptions like dyspnea are most commonly assessed using the imaging modalities of either PET (positron emission tomography) or fMRI (functional magnetic resonance imaging) which give a three-dimensional impression on the activity pattern derived from task dependent neuronal vascularization. For each voxel, the activity value is reported with the corresponding (x, y, z) coordinate which is used to compare the task with the baseline condition [8]. Oftentimes studies with PET and fMRI include only a limited sample size leading to low statistical power and reduced generalizability of the results. The method of ALE (activation likelihood estimation) meta-analysis is based on synthesizing the results of different studies investigating a comparable condition by combining the coordinates of activated voxels. Statistical significance is tested for the combined ALE map and clusters of activity are generated [9,10,11]. Since evidence on the functional neuroanatomy of dyspnea is limited, this work intends to synthesize the data of the existing literature by performing an ALE meta-analysis using the GingerAle software version 3.0.2.

## 2. Methods

**Figure 1:**
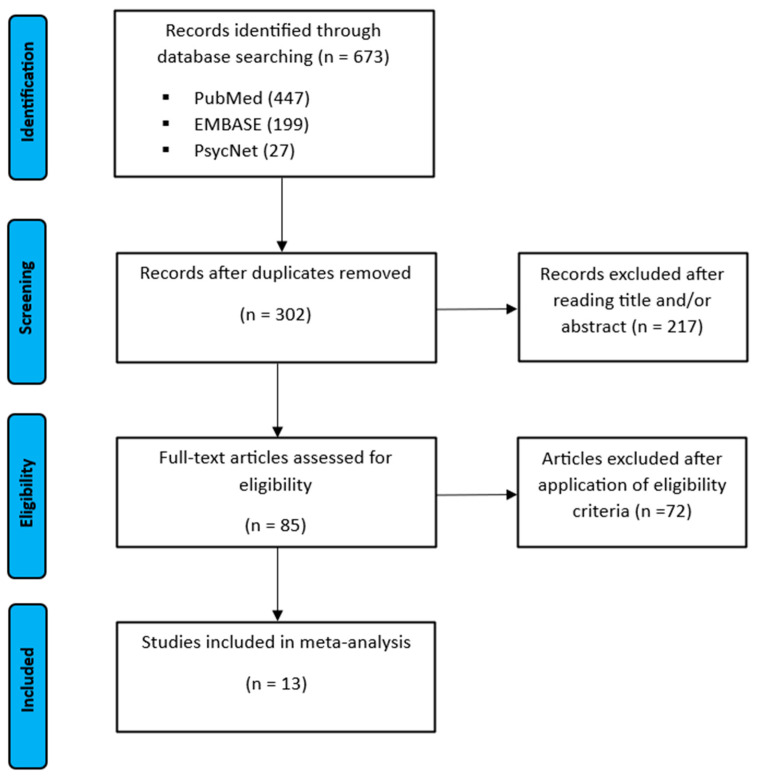
Flow chart of the study selection according to the Preferred Reporting Items for Systematic Reviews and Meta-Analyses (PRISMA).

The literature search was conducted according to the Preferred Reporting Items for Systematic Reviews and Meta-Analyses (PRISMA-P 2015) checklist as illustrated in Figure 1 [12].

### 2.1. Data Sources and Search Strategy

A comprehensive literature search was conducted across the databases PubMed, EMBASE and PsycNet until November 30 2024. The search strategy included the combination of each term with the Boolean operators AND and OR and search by proximity. The keywords and Medical Subject Heading (MeSH) terms “dyspnea” OR “air hunger” OR “breathlessness” AND “fMRI” OR “PET” in title and/or abstract. References were exported with the Citavi^©^ software version 7.0 (Lumivero, Denver, CO, USA) and duplicates were removed.

### 2.2. Eligibility Criteria

The final meta-analysis only included prospective interventional studies which investigated the neuroanatomical correlates of dyspnea perception induced by different laboratory stimuli including restrained ventilation, loaded breathing, and induced hypercapnia. Articles were excluded if they were reviews, letters or comments, editorials, case reports or case series and lacked information about the above-mentioned eligibility criteria or did not report the foci coordinates. No restrictions considering publication date or language of the published articles were applied.

### 2.3. Study Election and Data Extraction

The identified records were screened for eligibility by reading titles and abstracts of each article. If the eligibility criteria were met, the full text was obtained and investigated for inclusion in the meta-analysis. The x, y, z coordinates of each foci were extracted manually into a text file including the reference space, study name, condition and sample size. All foci data were converted to the MNI (Montreal Neurological Imaging) coordinate space before entering the calculation of the ALE meta-analysis. The within-condition analysis included all studies investigating the effect of different stimuli on brain activity using fMRI or PET scans. Subgroup analysis was performed for studies investigating restrained breathing and mechanical loading on neural correlates of dyspnea perception.

### 2.4. Statistical Analysis

The ALE meta-analysis was conducted using the software GingerALE version 3.0.2 (http://brainmap.org/ale/index.html) in an automated four step procedure. (1) At first, the foci data were entered into the GingerALE software which calculates the ALE values of each voxel using a 2 × 2 × 2 mm^3^ matrix. The ALE technique models the uncertainty of locations in a three-dimensional space as a Gaussian probability distribution centered about a peak at the reported coordinate. Using a random-effects model, a full-width half maximum (FWHM) depending on the subject size is applied to reflect the average smoothness of the input data. A wider FWHM is applied in data sets with a smaller subject size. Thereby a statistical map in which each voxel represents the probability that activation occurs is created. (2) The second step involves a permutation test to determine the statistical significance of each ALE value for which a random data set representing the null distribution at each voxel is calculated using a Monto Carlo simulation. The permutation test was run with 1000 permutations and an ALE map with a *p*-value for each voxel was determined. (3) A cluster-level inference was used to find clusters of data above a defined threshold. The ALE map was thresholded with a *p* < 0.05. (4) The cluster-forming threshold was *p* < 0.001 for a minimum cluster size of 100 mm^3^. The calculation of ALE maps was performed for all included studies to find the within-condition effect for the different conditions. In addition, ALE values were calculated for studies with a restrained breathing condition and those with a loaded breathing condition. The ALE maps were plotted on an anatomical MNI template using the Mango software version 4.1.

## 3. Results

### 3.1. Study Selection

The study selection process is illustrated in the flow chart in Figure 1. There was a total count of 673 records identified after the initial search including from 447 PubMed, from 199 EMBASE, and from 27 PsycNet. After removal of all duplicates, the remaining 673 records were then screened for eligibility. Finally, 13 studies were included in the meta-analysis. An overview on the characteristics of all included studies is provided in Table 1 and Table 2.

### 3.2. Within-Condition Analysis

**Figure 2:**
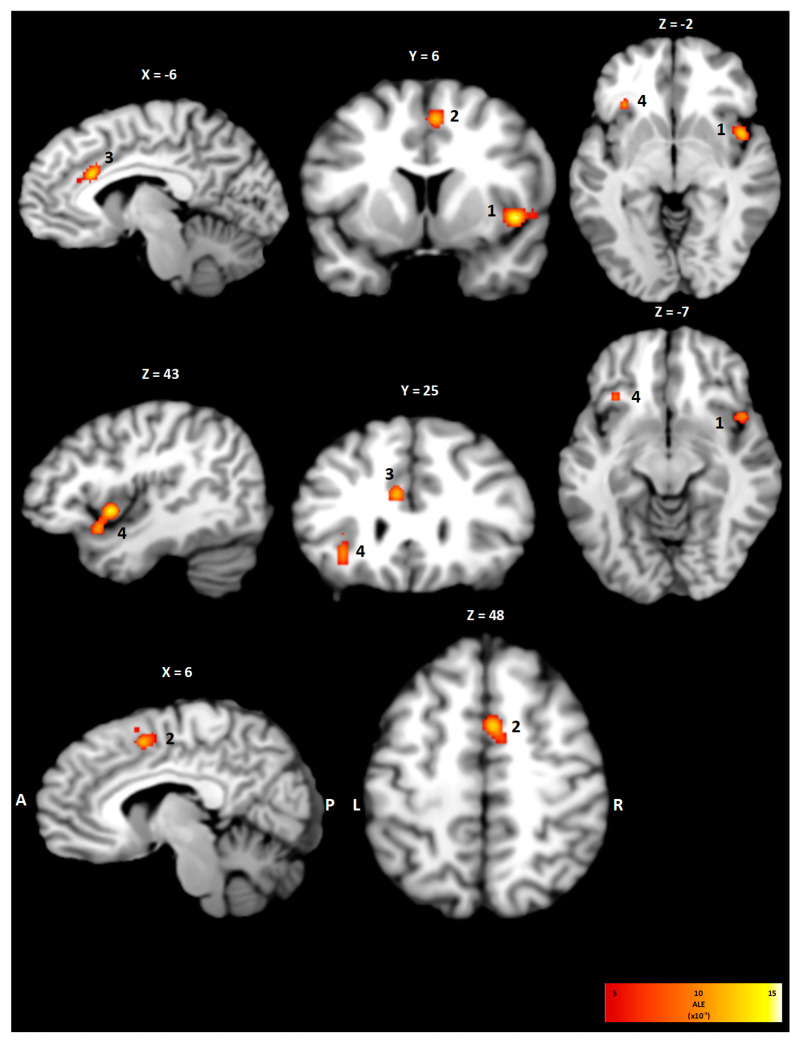
Clusters of the ALE meta-analysis for the within-condition in sagittal, coronal and transversal view. Statistically significant activation was found in both the right (1) and left (4) insula as well as for the right (2) and left (3) cingulate cortex. The ALE value of each voxel is color-coded according to the bar on the right.

The meta-analysis of all included studies investigating the neuroanatomical correlates of dyspnea resulted in four statistically significant clusters of activity as shown in Table 3 and Figure 2. On the right hemisphere a cluster of 1760 mm^3^ with coordinates for the weighted center in (44, 9, −4) involving the insula in Brodmann area (BA) 13 was observed. The right cingulate cortex showed a statistically significant activation with coordinates for the weighted center in (5, 6, 49) in BA 24. Correspondingly on the left hemisphere, clusters in the insula (744 mm^3^, −32, 23, 0) and in the cingulate cortex (888 mm^3^, −6, 23, 26) were observed.

### 3.3. Subgroup Analysis

**Figure 3:**
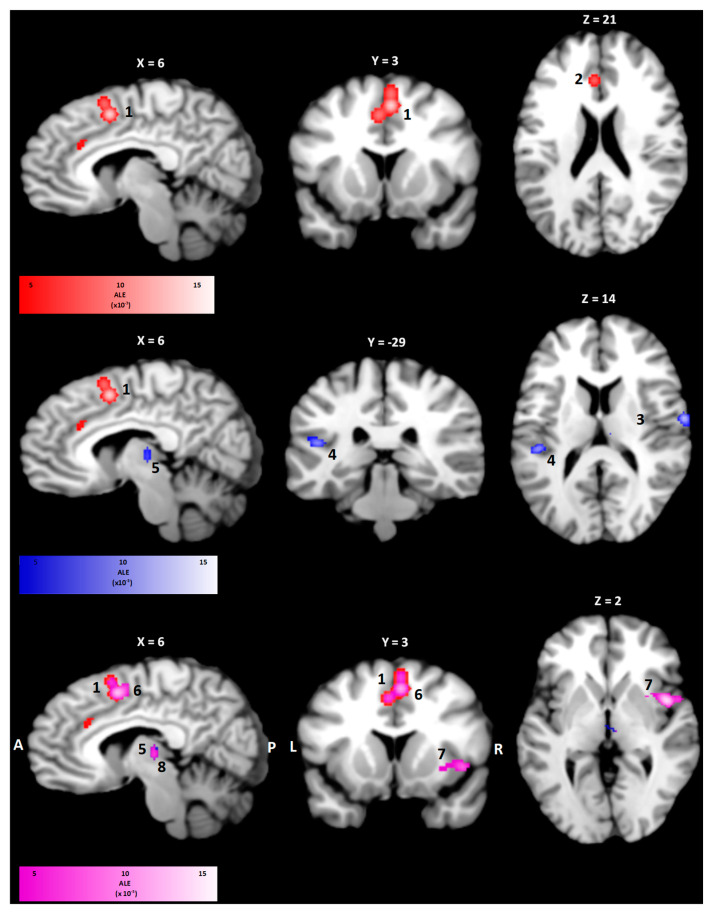
Clusters of the ALE meta-analysis for restrained breathing, loaded breathing and combined conditions in sagittal, coronal and transversal view. Statistically significant activation was found in both the right (1) and left (2) cingulate cortex for the restrained breathing condition. Clusters in the right postcentral gyrus (3), the left superior temporal gyrus (4) and the right thalamus (5) were observed for the loaded breathing condition. Combining ALE maps of both conditions, clusters in the right cingulate cortex (6), the right insula (7), and the right thalamus (8) were observed. The ALE value of each voxel is color-coded according to the bar below the images of each condition.

In order to the assess the brain activity related to either restrained or loaded breathing and to account for the heterogeneity among studies, separate ALE maps for both conditions were calculated as illustrated in Figure 3 and Table 4. For restrained breathing, statistically significant activity in the right and left cingulate cortex with peak coordinates in BA 24 (2448 mm^3^, 2, 20, 48) and in BA 32 (1296 mm^3^, −3, 28, 23) were observed. The loaded breathing condition led to clusters in the right postcentral gyrus (BA 1, 560 mm^3^, 63, −7, 14), the left superior temporal gyrus (BA 41, 512 mm^3^, −47, −30, 15) and the right thalamus (504 mm^3^, 5, −17, 7). The combined activity pattern of both conditions showed clusters in the right cingulate cortex (BA 24, 1800 mm^3^, 4, 8, 48), the right insula (BA 13, 1376 mm^3^, 10, −17, 8), and the right thalamus (688 mm^3^, 10, −17, 8).

## 4. Discussion

This ALE meta-analysis demonstrates the neuroanatomical correlates of dyspnea by synthesizing studies using either PET or fMRI during different breathing challenges. The cluster analysis for all included studies showed statistically significant activation for the insula and ACC in both hemispheres. Subgroup analysis for the restrained and loaded breathing condition revealed a divergent activity pattern involving the sensory and affective pathway of dyspnea perception [26]. While the loaded breathing condition showed clusters involving the postcentral gyrus, the superior temporal gyrus, and the thalamus, restrained breathing challenges led to statistically significant activation in the right and left cingulate cortex.

These findings substantiate the idea of two distinct pathways underlying the sensory and affective domain of dyspnea perception [27]. The intensity or sensory quality of breathlessness is related to afferents from mechanoreceptors of respiratory muscles which project to the brainstem medulla and are relayed in the ventroposterior thalamus to the primary and secondary somatosensory cortex. This discriminative pathway of respiratory proprioception encodes the intensity level of dyspnea or work of breathing and involves projections to motor areas from where efferent commands are sent to the respiratory muscles.

The affective pathway includes parts of the limbic system and represents the qualitative domain of dyspnea perception which is often referred to as air hunger [26,27]. It begins with respiratory vagal afferents which project to the brainstem medulla from where projections are sent to the amygdala and mediodorsal areas of the thalamus. This pathway further includes the insula and cingulate cortex which are interconnected and represent the emotional or qualitative domain of dyspnea. Both the insula and cingulate cortex are subdivided into areas which are characterized by specific functions of sensorimotor integration. The insula shows a posterior to mid-anterior pattern of information integration and is functionally subdivided into four distinct regions [28]. The anterior-ventral part of the insula is connected to the ACC and represents the socio-emotional domain of dyspnea perception. The anterior-dorsal insula is involved in cognitive tasks and connected with the anterior cingulate and the parietal cortex. While the central insula is engaged in olfactogustatory perception, the mid-posterior region of the insula is related to sensorimotor integration and derived reactions to the perceived sensory input. Both the insula and the cingulate cortex are not only part of the limbic system, but represent visceromotor areas (VMA) which are involved in assessing interoceptive states to regulate autonomic homeostasis [29,30].

The cingulate cortex is divided into four posterior to mid-anterior regions with specific functional organization. The ACC is related to emotion salience and visceral integration and is connected to structures of the ventral or “what” stream of sensorimotor integration [31]. It represents interoceptive states which guide actions towards or away from a perceived goal [32]. The medial cingulate cortex is involved in response selection, is connected with the posterior cingulate cortex (PCC), and is related to visuospatial orientation. The retrosplenial cortex is connected to hippocampal areas and contributes to memory formation.

In general, the ACC and PCC are associated with distinguishable tasks of interoceptive or spatio-temporal processing and are related to either the ventral “what” or dorsal “where stream. The ACC is connected with structures of the limbic system including the orbitofrontal cortex, the amygdala and the anterior insula which encode the emotional quality of perceived stimuli. As shown for the within-condition ALE map, a co-activation of the anterior insular cortex and the ACC is regularly observed. In addition, a linear activation of both the AIC and the ACC with the (un-) pleasantness of the perception has been demonstrated [33].

Regarding the results of the ALE meta-analysis, the bi-hemispheric activation of the insula and cingulate cortex for all included studies, therefore, substantiates the idea of a qualitative affective pathway of dyspnea perception. This finding may be clinically most evident with fear being the most commonly reported emotion associated with breathlessness [34]. In contrast, the posterior part of the cingulate cortex which is connected with the dorsolateral prefrontal cortex and parietal areas engaged in executive control. It is part of the dorsal “where stream of sensorimotor integration and is less activated during perceived breathlessness”.

While the affective and sensory pathway encode distinguishable qualities of dyspnea which underly distinct anatomical substrates, both share the common goal of correcting for autonomic disbalances to maintain visceral homeostasis. The awareness of regularly unconscious states is referred to as interoception and can be embedded within the broader concepts of predictive coding and active inference [35,36]. Accordingly, VMA (e.g., the ACC) generate predictions about future sensory events which are compared with the actual sensory feedback. A mismatch between these predictions and the feedback from vagal and somatosensory afferents causes a prediction error which leads to the emerging sensation, for example, dyspnea. This is structurally organized within visceromotor afferents which not only send motor commands to respiratory muscles but provide an efference copy for viscerosensory areas [37]. In case of a mismatch, predictions generated by VMA are updated to match the actual sensory feedback. This process of neuronal adaptation referred to as predictive coding may explain the activation in the insula und cingulate cortex which provide homeostatic set-points against which the feedback of vagal afferents is compared [38]. In addition to the emergence of perceived dyspnea, the prediction error induces a response of the effector system to correct for the detected disbalance. This control and regulation system includes an autonomic reflex circuit for the affective pathway and projections to higher motor areas for the somatosensory system. Theoretically, the emergence of a behavioral response is related to the framework of active inference which states that prediction errors cause the effector system to seek for a resolution of the occurred mismatch [39]. Therefore, the awareness of usually unconscious inner states (interoception) can be regarded as a basic from of consciousness which prompts the individual to adapt its behavior towards regaining autonomic homeostasis. Accordingly, the activation of the insula is related to the mental experience of inner states (feelings), to which the ACC attributes a motivational value thereby influencing the individual’s behavior on an emotional level and inducing an adaptive response [40].

Besides the immediate consequences on perception and behavior, the affective pathway may also contribute to memory formation by sending input into the hippocampal memory system via afferents from the anterior cingulate to the peri-/entorhinal cortex [41]. This may explain the subjectivity of dyspnea perception which is cognitively and emotionally modulated based on previous experiences. In addition, the concept of gain control in interoceptive inference relates to the finding that the emergence of sensations is influenced by the current emotional and attentional state which may or may not contribute to the awareness of a mismatch between predictions and sensory feedback [38]. The present work, therefore, contributes to an improved understanding of the neuroanatomical correlates underlying the interindividual differences in dyspnea perception.

## 5. Conclusions

This ALE meta-analysis demonstrates that the sensation of dyspnea can be described on an emotional and intensity level depending on the applied breathing challenge. These perceptual qualities are related to distinct neuroanatomical substrates that allow for the interoception of usually unconscious states and thereby enable the individual to adapt its behavior towards regaining autonomic homoeostasis. Both the conscious perception of dyspnea and the behavioral response can be explained with the framework of predictive coding and active inference. In addition, the observed neuroanatomical correlates of breathlessness involving parts of the limbic and the somatosensory system substantiate the clinically observed individual differences in dyspnea perception which is based on previous experiences and the current emotional and attentional state.

## Figures and Tables

**Table 1:** Studies included in the meta-analysis.

Study ID			Population			Intervention	
Author (Year)	Journal	Study Design	Imaging Modality	Sample Size (% Female)	Age (Years)	Conditions	Foci
Banzett et al. (2000) [13]	*Brain Imaging NeuroReport*	Prospective interventional study	PET	8 (0)	n.a.	Ventilation via nose mask or mouthpiece by positive pressure ventilation controlling for pCO_2_ constant at 40 Torr, 14 breaths/minute	13
Liotti et al. (2000) [14]	*PNAS*	Prospective interventional study	PET	8 (n.a.)	n.a.	Administration of 8% CO_2_/92% O_2_ via face mask or mouth piece, dyspnea rating scale from 0 to 100	14
Pfeiffer et al. (2001) [15]	*Am J Respir Crit Care Med*	Prospective interventional study	PET	8 (0)	33 ± 3 (mean ± SD)	Two loads that induce a sensation of either slight/moderate or severe dyspnea in more than 50% of runs, 10 s before data acquisition the load was added	10
Evans et al. (2002) [16]	*Journal of Neurophysiology*	Prospective interventional study	fMRI (2 T)	6 (4)	25–32 (range)	13.2 breaths/min, pCO_2_ constant by raising inspired CO_2_, low V_t_ = 0.75 ± 0.21 L, high V_t_ = 1.38 ± 0.06 L	23
Macey et al. (2006) [17]	*Resp Physiol* *Neurobiol*	Prospective interventional study	fMRI (1.5 T)	7 (0) OSAS, 12 (0) controls	46 ± 5 (mean ± SD) 47 ± 3 (mean ± SD)	Nose clip and breathing via mouthpiece, each trial of 60 s unrestricted breathing, 90 s of inspiratory loading (−6 to 15 mmHg)	11
McKay et al. (2008) [18]	*Neuroimage*	Prospective interventional study	fMRI (2 T)	8 (6)	20–35	Breath holds for 15 s at resting expiratory lung volume, raised inspiratory CO_2_ for 15 s to increase _pet_CO_2_ by 7–8 mmHg	19
von Leupoldt et al. (2008) [19]	*Am J Respir Crit Care Med*	Prospective interventional study	fMRI (3 T)	14 (7)	26.6 ± 6.2 (mean ± SD)	11 conditions of inspiratory loaded and unloaded breathing continuous for 24 s with a mean load of 8.22 kPa/L/s, loaded condition with dyspnea pictures	9

Abbreviations: Am J Respir Crit Care Med, American Journal of Respiratory and Critical Care Medicine; fMRI, functional magnetic resonance imaging; n.a., not available; OSAS, obstructive sleep apnea syndrome; PET, positron emission tomography; PNAS; Proceeding of the National Academy of Science, Resp Physiol Neurobiol; Respiratory Physiology & Neurobiology; SD, standard deviation, T, Tesla, V_t_, tidal volume.

**Table 2:** Studies included in the meta-analysis.

Study ID			Population			Intervention	
Author (Year)	Journal	Study Design	Imaging Modality	Sample Size (% Female)	Age (Years)	Conditions	Foci
Binks et al. (2014) [20]	*Resp Physiol* *and Neurobiol*	Prospective interventional study	fMRI (1.5 T)	8 (3)	23–31 (range)	Mechanical ventilation with constant mild hypercapnia (~45 mmHg), V_t_ alternating between 0.96 ± 0.23 L and 0.48 ± 0.08 L, air hunger on transient and steady state	9
Herigstad et al. (2015) [21]	*Chest*	Prospective interventional study	fMRI (3 T)	41 (15) COPD 40 (16) controls	68.0 ± 8.2 (mean ± SD)	Dyspnea related cues, rating on a scale from 0 to 100	13
Esser et al. (2017) [22]	*Frontiers in Physiology*	Prospective interventional study	fMRI (3 T)	17 (8) in COPD 21 (11) in controls	65.6 ± 9.3 (mean ± SD) in COPD 63.4 ± 8.8 (mean ± SD) in controls	Inspiratory resistive loads of increasing magnitude, 10 blocks of mild dyspnea (smallest load), 10 blocks of severe dyspnea (≥5 of 10 on Borg Scale) each lasting for 24 s	13
Chan et al. (2018) [23]	*Frontiers in Physiology*	Prospective interventional study	fMRI (3 T)	23 (13)	23.7 ± 3.1 (mean ± SD)	Inspiratory occlusion of 150 ms at the onset of inspiration every 2–4 breaths, 12 min of acquisition time, at least 32 successfully occluded breaths, rating of level of breathlessness based on VAS from 0 to 100	8
Hassanpour et al. (2018) [24]	*Neuropharmacology*	Prospective interventional study	MRI (3 T)	22 (11)	26 ± 6 (mean ± SD)	Intravenous infusion of isoproterenol hydrochlorid and normal saline as control, each dose (1 μg, 2 μg and normal saline) was repeated twice, intensity of breathing sensation (0 = none, 10 most ever) was reduced	7
Chan et al. (2019) [25]	*Scientific Reports nature research*	Prospective interventional study	MRI (3 T)	34 (20)	23 ± 3.4 (mean ± SD)	Breathing through face mask while respiration was repeatedly interrupted by inspiratory occlusion of 150 ms delivered every 2–4 breaths, at least 32 breaths were collected	14

Abbreviations: COPD, chronic obstructive pulmonary disease; fMRI, functional magnetic resonance imaging; Resp Physiol Neurobiol; Respiratory Physiology & Neurobiology; SD, standard deviation; T, Tesla, V_t_, tidal volume.

**Table 3:** Location of clusters with statistically significant brain activation and coordinates for the weighted center for all included studies.

Cluster	Brain Region	BA	x	y	z	Volume (mm^3^)	ALE (×10^−3^)
1	Right Insula	13	44	9	−4	1760	18.7
2	Right Cingulate Cortex	24	5	6	49	976	17.4
3	Left Cingulate Cortex	24	−6	23	26	888	17.4
4	Left Insula	13	−32	23	0	744	15.2

Abbreviations: ALE, activation likelihood estimation; BA, Brodmann area.

**Table 4:** Location of clusters with statistically significant brain activation and weighted center for the restrained and loaded breathing condition.

Cluster	Brain Region	BA	x	y	z	Volume (mm^3^)	ALE (×10^−3^)
Restrained breathing							
1	Left/Right Cingulate Cortex	24	2	10	48	2448	16.9
2	Left Cingulate Cortex	32	−3	28	23	1296	11.0
Loaded Breathing							
3	Right Postcentral gyrus	1	63	−7	14	560	13.4
4	Left Superior Temporal Gyrus/Transverse Temporal Gyrus	41	−47	−30	15	512	12.0
5	Right Thalamus	-	5	−17	7	504	9.9
Restrained and Loaded Breathing							
6	Right Cingulate Cortex	24	4	8	48	1800	17.4
7	Right Insula	13	43	6	1	1376	18.5
8	Right Thalamus		10	−17	8	688	11.3

Abbreviations. ALE, activation likelihood estimation; BA, Brodmann area.

## Data Availability

All data generated or analyzed during this study are included in this article. Further inquiries can be directed to the corresponding author.

## Abbreviations

ACC	Anterior cingulate cortex
ALE	Activation likelihood estimation
BA	Brodmann area
fMRI	Functional magnetic resonance imaging
FWHM	Full width half maximum
MNI	Montreal Neurological Imaging
PCC	Posterior cingulate cortex
PRISMA	Preferred Reporting Items for Systematic Reviews and Meta-Analyses
VMA	Visceromotor areas
